# Cytotoxic *Escherichia coli* strains encoding colibactin and cytotoxic necrotizing factor (CNF) colonize laboratory macaques

**DOI:** 10.1186/s13099-017-0220-y

**Published:** 2017-12-06

**Authors:** Yan Feng, Anthony Mannion, Carolyn M. Madden, Alton G. Swennes, Catherine Townes, Charles Byrd, Robert P. Marini, James G. Fox

**Affiliations:** 1 Division of Comparative Medicine, Massachusetts Institute of Technology, 77 Massachusetts Avenue, 16-825, Cambridge, MA 02139 USA; 2 Present Address: Center for Comparative Medicine, Baylor College of Medicine, Houston, TX USA; 3 Present Address: North Powers Animal Hospital, Colorado Springs, CO USA

## Abstract

**Background:**

Many *Escherichia coli* strains are considered to be a component of the normal flora found in the human and animal intestinal tracts. While most *E. coli* strains are commensal, some strains encode virulence factors that enable the bacteria to cause intestinal and extra-intestinal clinically-relevant infections. Colibactin, encoded by a genomic island (*pks* island), and cytotoxic necrotizing factor (CNF), encoded by the *cnf* gene, are genotoxic and can modulate cellular differentiation, apoptosis and proliferation. Some commensal and pathogenic *pks*+ and *cnf*+ *E. coli* strains have been associated with inflammation and cancer in humans and animals.

**Results:**

In the present study, *E. coli* strains encoding colibactin and CNF were identified in macaque samples. We performed bacterial cultures utilizing rectal swabs and extra-intestinal samples from clinically normal macaques. A total of 239 *E. coli* strains were isolated from 266 macaques. The strains were identified biochemically and selected isolates were serotyped as O88:H4, O25:H4, O7:H7, OM:H14, and OM:H16. Specific PCR for *pks* and *cnf1* gene amplification, and phylogenetic group identification were performed on all *E. coli* strains. Among the 239 isolates, 41 (17.2%) were *pks*+*/cnf1*−, 19 (7.9%) were *pks*−/*cnf1*+, and 31 (13.0%) were *pks*+/*cnf1*+. One hundred forty-eight (61.9%) *E. coli* isolates were negative for both genes (*pks*−/*cnf1*−). In total, 72 (30.1%) were positive for *pks* genes, and 50 (20.9%) were positive for *cnf1*. No *cnf2*+ isolates were detected. Both *pks*+ and *cnf1*+ *E. coli* strains belonged mainly to phylogenetic group B2, including B2_1_. Colibactin and CNF cytotoxic activities were observed using a HeLa cell cytotoxicity assay in representative isolates. Whole genome sequencing of 10 representative *E. coli* strains confirmed the presence of virulence factors and antibiotic resistance genes in rhesus macaque *E. coli* isolates.

**Conclusions:**

Our findings indicate that colibactin- and CNF-encoding *E. coli* colonize laboratory macaques and can potentially cause clinical and subclinical diseases that impact macaque models.

**Electronic supplementary material:**

The online version of this article (10.1186/s13099-017-0220-y) contains supplementary material, which is available to authorized users.

## Background

*Escherichia coli* is the predominant aero-anaerobic Gram-negative species of the normal microflora colonizing the gastrointestinal tract of humans and animals [1]. Most *E. coli* strains are commensal and rarely cause clinically-relevant disease. However, some strains carry virulence genes that enable selected *E. coli* strains to cause intestinal and extra-intestinal infections [2, 3]. Based on a phylogenetic assay, *E. coli* can be classified into four main phylogenetic groups (A, B1, B2 and D) [4–7]. Pathogenic strains which often encode virulence factors belong to group B2 and D, while most fecal *E. coli* strains belonging to group A and B1 lack virulence factors [8, 9]. A group of cytotoxins, including colibactin, cytotoxic necrotizing factors (CNFs), cytolethal distending toxin (CDT) and cycle inhibiting factor (CIF), are classified as cyclomodulins and are genotoxic and/or modulate cellular differentiation, apoptosis and proliferation [10–12].

Colibactin is a cytotoxic hybrid polyketide/nonribosomal peptide produced by several species of *Enterobacteriaceae*. It was first identified in 2006 in an extra-intestinal pathogenic *E. coli* (ExPEC) strain isolated from a case of neonatal meningitis [12]. This secondary metabolite, colibactin, is produced by the *clbA*-*S* genes present in the 54-kb pathogenicity *pks* island, a genetic island encoding a non-ribosomal peptide synthetase-polyketide synthase (NRPS-PKS) assembly line [12, 13]. In vitro studies have shown that *pks*+ *E. coli* strains induce enlargement of cells and nuclei without mitosis (megalocytosis), cause G2 cell cycle arrest, and DNA double strand breaks [12]. In animal model experiments, a *pks*+ *E. coli* strain (NC101), isolated from specific pathogen free wild-type mice induced inflammation in the cecum in interleukin 10 knockout (IL10^−/−^) mice after a 3 week monoassociation period [14]. Studies also demonstrated that monoassociation with NC101 promotes invasive carcinoma in IL10^−/−^ mice treated with azoxymethane (AOM). The promotion effect was dependent on expression of the *pks* island [15]. In a previous study from our laboratory, 88% of *E. coli* isolates from laboratory mice encoded *pks* genes and belonged to phylogenetic group B2 [16]. Our findings indicated that colibactin-encoding *E. coli* commonly colonize laboratory mice and may induce clinical and subclinical disease that may impact in vivo experimental results [16].

*Escherichia coli* strains that produce CNF belong to the pathotype necrotoxigenic *E. coli* (NTEC) and are associated with intestinal and extra-intestinal infection in both humans and animals [2]. The majority of CNFs include chromosomally encoded *cnf1* [17] and plasmid-encoded *cnf2* [18]. CNF1 is a 115 kDa protein toxin which activates Rho GTPases, leading to cytoskeletal and cell cycle alterations with subsequent macropinocytosis and formation of megalocytic, multinucleated cells. CNF1-producing and β-hemolytic *E. coli* strains most notably cause urinary tract and meningeal infections in humans [19]. These strains are also isolated from healthy and diseased animals. In our laboratory, *cnf1*+ *E. coli* strains were isolated from ferrets with diarrhea and extra-intestinal infections [20] and from healthy macaques [21]. *cnf1*-encoding *E. coli* strains have also been isolated from cats [22], dogs [23, 24], pigs [25], and birds [26].

The prevalence of *pks*+ *E. coli* in rhesus macaques is not known, nor is there published evidence that *E. coli* strains encoding both *pks* and *cnf* genes colonize macaques. The purpose of the present study was to characterize rectal *E. coli* isolates, as well as extra-intestinal isolates, from macaques for the presence of specific virulence genes (*pks* and *cnf*) and demonstrate their in vitro toxin activities.

## Methods

### Animals

Macaques (*Macaca mulatta* and *M. fascicularis*), originally received from three US-based vendors, received physical examinations and routine diagnostic evaluations during quarantine and at quarterly intervals in 2012, 2014 and 2016. The colony contained 84 animals in 2012 (4 cynomolgus macaques and 80 rhesus macaques, of which 23 were female), 85 animals in 2014 (4 cynomolgus macaques and 81 rhesus macaques, of which 24 were female) and 97 animals in 2016 (2 cynomolgus macaques and 95 rhesus macaques, of which 25 were female). They ranged in age from 4 to 20 years. Animals were routinely pair-housed and maintained in an animal facility accredited by the Association for Assessment and Accreditation of Laboratory Animal Care (AAALAC) International. They were fed specified amounts of primate chow (Purina Lab Diet 5038) twice a day and supplemented daily with treats, seasonal fruits and vegetables. Water was provided ad libitum when animals were not on studies requiring water regulation. Housing conditions were maintained at 20–22 °C, 30–70  % humidity, 10–15 non-recirculated air changes per hour and a light cycle of 12 h light:12 h dark.

### Microbiological analysis

#### Culture and isolation

Two hundred and sixty-nine samples (265 rectal swab, one gingival, two from cephalic recording chambers and one from margin skin) were collected from a cohort of 266 clinically normal macaques (those received in quarantine as well as the established cohort of macaques actively being used in neurobiological research) during quarterly colony health examinations in 2012 (n = 84), 2014 (n = 85) and 2016 (n = 97). Rectal swabs were placed in tubes with sterile Gram-negative broth (BD) and incubated aerobically overnight at 37 °C. The next day, a sterile swab was placed in the Gram-negative culture tube and then streaked onto MacConkey lactose agar plates (BD/BBL, Sparks, MD). For extraintestinal samples, the swab were placed in sterile trypticase soy broth (TSB) (BBL) and incubated aerobically overnight at 37 °C. The next day, a sterile swab was placed in the TSB culture tube and streaked onto Chocolate agar (BBL) and a split plate of blood agar with sheep blood and MacConkey agar (BBL). Lactose-positive colonies were selected and plated onto a sheep blood agar plate (Remel, Lenexa, KS), incubated aerobically, and β-hemolysis, if present, was noted. Suspect *E. coli* isolates were then characterized by analytic profile index (API) 20 E (Biomérieux, Cambridge, MA). API 20 E is a panel of biochemical tests used for the identification and differentiation of enteric Gram-negative rods. A profile number, determined by the sequence of positive and negative test reactions, is referenced in the API codebook database to determine the bacterial species. Swabs from cranial implants were placed in sterile TSB (BBL), used as an enrichment broth. The swabs were then plated onto chocolate agar (BBL) and a split plate of blood agar with sheep blood and MacConkey agar (BBL). Colonies that were lactose-fermenting positive and Gram-negative on Gram’s stain were subcultured onto sheep blood agar and identification was confirmed with the API 20 E (Biomérieux).

#### Clinical correlation

Medical records were evaluated for temporal correlations of culture and clinical signs from animals which had *pks*+ or* pks*/*cnf1* double-positive* E. coli* isolates. Four records (3 for 2012 and 1 for 2014) were unavailable.

#### DNA extraction, PCR amplification, and sequencing

*Escherichia coli* colonies grown on blood agar plates were collected in sterile phosphate buffered saline (PBS) in microfuge tubes, boiled for 10 min, followed by centrifugation at 12,000*g* for 10 min. The supernatant was used for PCR analysis. The primers and annealing temperatures used are shown in Additional file 1: Table S1. To detect the colibactin genes, three sets of primers were used to amplify *clbA, clbB* and *clbQ* genes. Multiplex *cnf* primers were used to screen for the *cnf* gene followed by PCR with *cnf1* and *cnf2* specific primers to further distinguish *cnf* subgroups. Selected strains were assayed for *cdt* and *cif* using primers listed in Additional file 1: Table S1. To determine the phylogenetic groups of isolates, five sets of primers for the genes *yjaA, TspE4.C2, chuA, svg* and *uidA* were used in a multiplex PCR [4, 7]. The phylogenetic groups were determined based on the PCR gel pattern. Sequencing of 16S rRNA and *clbA* and *clbQ* genes was performed at QuintaraBio (Allston, MA) using primers 9F and F16 (16s rRNA), IHAPJPN42 and IHAPJPN46 (*clbA*), IHAPJPN55 and IHAPJPN56 (*clbQ*) for selected isolates.

#### Serotyping

Eleven *E*. *coli* isolates chosen from different cohorts and representing *pks*+/*cnf1*−, *pks*−/*cnf1*+, *pks*+/*cnf1*+, and *pks*−/*cnf1*− genotypes were submitted to the *E*. *coli* Reference Center at Penn State University for serotype testing, which included: O and H typing and PCR analyses for heat-labile enterotoxin (*elt*), heat-stabile enterotoxin (*estA* and *estB*), Shiga-type toxin 1 and 2 (*Stx1* and *Stx2*), intimin gamma (*eae*), *cnf1*, and *cnf2*.

#### Cytotoxicity assay

*Escherichia coli* strains screened in the cytotoxicity assay included K12 (negative control), V27 (a *pks* and *cdt* positive control acquired from the *E*. *coli* Reference Center), NC101 and NC101∆*pks* (a *pks* positive control and *pks* mutant strain, respectively, both gifts from Dr. Christian Jobin), and 12 selected *E. coli* isolates representing *pks*+/*cnf1*−, *pks*−/*cnf1*+, *pks*+/*cnf1*+, and *pks*−/*cnf1*− genotypes (three for each genotype). Selected tests were also performed on the *E*. *coli* strains used in the cytotoxicity assays and included: API 20 E for biochemical characterization and PCR for phylogenetic groups as well as for *clbA*, *clbQ*, *cnf1*, *cif* and *cdt* genes.

#### Cell culture assay for colibactin cytotoxicity

The cytotoxicity assay was performed as described previously with modifications [12, 16]. HeLa S3 cells (ATCC CCL2.2) were grown and maintained in Eagle’s Minimum Essential Medium (EMEM, ATCC) containing 10% Fetal Calf Serum (FCS, Sigma) and 1% antibiotic–antimycotic (Gibco) at 37 °C with 5% CO_2_. 5 × 10^3^ cells were seeded onto 96-well cell culture plates and incubated at 37 °C with 5% CO_2_ for 24 h. Overnight liquid cultures of *E*. *coli* strains were grown for 2 h at 37 °C and then adjusted to O.D. 600 nm in 1% FCS EMEM media to concentrations corresponding to a multiplicity of infection (MOI; the number of bacteria per cell at the onset of infection) of 1, 5, 25 and 100, respectively. Following inoculation, plates were centrifuged at 200*g* for 10 min to facilitate bacterial interaction and then incubated at 37 °C with 5% CO_2_ for 4 h. Cells were then washed with EMEM and replaced with EMEM containing 10% FCS and 200 µg/mL gentamicin (Gibco). Following 72 h incubation, plates were stained with Diff-quick stain (Thermo Scientific). Cells were then inspected under a microscope for confluence and morphological changes. Images were captured with a Zeiss Axiovert-10 microscope using Image Pro-Plus software version 7.0 at 20× magnification.

#### Cell culture assay for sonicate cytotoxicity

Overnight cultures of *E*. *coli* strains were pelleted by centrifugation at 12,000 rpm for 5 min. Supernatant was removed, filtered through a 0.2 μm filter, and stored at − 80 °C for later use. The pellets were washed in 1 mL of PBS and pelleted again by centrifugation at 12,000 rpm for 5 min. Pellets were re-suspended in 2 mL of PBS and then sonicated on ice using the following program: amplitude: 35; power: 7 W; 30 s intervals for a total of 5 min with 1 min breaks between intervals. Sonicate samples were centrifuged at 12,000 rpm for 10 min at 4 °C to remove large debris and filtered through a 0.2 µM filter. Total protein was quantified using the BCA assay (Thermo Fisher Scientific). HeLa cells, 5 × 10^3^ were seeded onto 96-well, cell culture plates and incubated at 37 °C with 5% CO_2_ for 24 h. Cells were treated with crude bacterial sonicate (40 μg/mL total protein) or supernatant (25 μL) for 72 h. Cells were stained and microscopically analyzed for confluence and morphological changes, as described above.

#### Draft genome sequencing and comparative analysis

The draft genomes of ten representative rhesus macaque *E. coli* isolates were sequenced. Genomic DNA was isolated using the MasterPure Complete DNA and RNA Purification Kit (Epicentre, Madison, WI) following the manufacturer’s protocol for bacterial cell samples. DNA libraries were prepared by the Sequencing Core at the Forsyth Institute (Cambridge, MA) using NextraXT for sequencing of 2 × 150 paired-end reads by Illumina MiSeq. Raw sequenced reads were decontaminated of adapter sequences and quality trimmed to a Phred quality score (Q) ≥ 10 using BBDuk from the BBMap package version 37.17 (http://sourceforge.net/projects/bbmap/). Decontaminated reads were then assembled into contigs with SPAdes followed by genome annotation with RAST, both services hosted by PATRIC [27]. Sequences encoding putative virulence factor and antibiotic-resistance genes were identified using VirulenceFinder 1.5 [28] and ResFinder 2.1 [29], both using the 90% identity and 60% minimum length threshold parameters. Syntenic relationships of *pks* genes and the hemolysin-*cnf1* operon between genomes were determined with SimpleSynteny [30].

## Results

### Clinical correlates

Evaluation of medical records did not establish an unequivocal association between *E. coli*—culture status and clinical signs. There were several animals who had experienced soft feces in the months before rectal culture, but the presence of other agents (e.g. *Balantidium coli* or *Trichuris trichuria*) or clinical conditions (e.g. gastric ulcer and *Helicobacter suis* gastritis) preclude definitive statements. *E. coli* was isolated from feces in the vast majority of cases, but *E. coli* isolates were also obtained from gingiva and surgical implants. In one *E. coli* positive animal, a severe eosinophilic colitis was identified via colonoscopy and histopathological evaluation in an appropriate time frame to suspect *E. coli*-associated colitis, but this animal also had immune-mediated thrombocytopenia and harbored *Balantidium coli* [31].

### Microbiological characterization of *E. coli* strains isolated from macaques

A total of 239 *E. coli* isolates were cultured from 269 rectal swabs and extraintestinal sites obtained from 266 macaques. The yearly prevalence of *E. coli* in macaques was 60.7% (51 out of 84), 85.7% (72 out of 84) and 75.3% (73 out of 97) in years 2012, 2014, and 2016, respectively (Table 1). In some macaques, there were more than one isolates as determined by different colony morphology characteristics, hemolysis and API code. Forty-three (18.0%) of 239 *E. coli* isolates were positive for β-hemolysis on sheep blood agar plates (Table 1). The most common API code recorded in this study was 5144572 observed in 44.8% (107 out of 239) of *E. coli* isolates. Other API codes assigned to the *E. coli* isolates were 1144572 (11.0%), 5044552 (7.5%), 5144552 (6.9%), 5044542 (6.4%) and 5144512 (4.0%). Fewer than 1% of isolates belonged to other API codes. These API codes confirm the isolates cultured were *E. coli*.

**Table 1:** Prevalence of *E. coli* strains isolated from macaques

	2012	2014	2016	Total
*E. coli* positive animals	51/84 (60.7%)	72/85 (85.7%)	73/97 (75.3%)	196/266 (73.7%)
β-hemolytic *E. coli* isolates	4/66 (6.1%)	16/84 (19.0%)	23/89 (25.8%)	43/239 (18.0%)

### *Escherichia coli* isolates from macaques encoding colibactin and CNF1

**Fig. 1:**
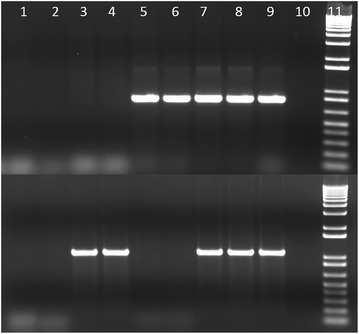
Amplification of *clbQ* and *cnf1* genes in *E. coli* isolates from macaques. Top row: *clbQ* gene, bottom row: *cnf1* gene. Lanes 1 and 2, S7 and S8 (*pks*-/*cnf1*−); lanes 3 and 4, S9 and S10 (*pks*−/*cnf1+*); lanes 5 and 6, S4 and S5 (*pks*+/*cnf1*−); lanes 7 and 8, S1 and S2 *(pks*+/*cnf1*+); lane 9, *pks* and *cnf1* positive controls; lane 10, negative controls; lane 11, 1 kb+ molecular marker

To detect whether cytotoxic virulence factors were present in isolated *E. coli,* PCRs were performed with primers for the *clbA*, *clbB* and *clbQ* genes of the *pks* island, and the *cnf1*and *cnf2* genes. Among the 239 *E. coli* isolates, 41 (17.2%) were *pks*+/*cnf1*−, 19 (7.9%) were *pks*−/*cnf1*+, and 31 (13.0%) were *pks*+/*cnf1*+. One hundred forty-eight (61.9%) *E. coli* isolates were *pks*−/*cnf1*− (Table 2, Fig. 1). No *cnf2*+ isolates were detected.

**Table 2:** Prevalence of *pks* and *cnf1* genes in *E. coli* isolates from macaques

	2012	2014	2016	Total
*pks*+/*cnf1*− (A)	11/66 (16.7%)	22/84 (26.2%)	8/89 (9.0%)	41/239 (17.2%)
*pks*−/*cnf1*+ (B)	0/66 (0.0%)	5/84 (6.0%)	14/89 (15.7%)	19/239 (7.9%)
*pks*+/*cnf1*+ (C)	7/66 (10.6%)	14/84 (16.7%)	10/89 (11.2%)	31/239 (13.0%)
Total *pks*+ (A + C)	18/66 (27.2%)	36/84 (42.9%)	18/89 (20.2%)	72/239 (30.1%)
Total *cnf1*+ (B + C)	7/66 (10.6%)	19/84 (22.6%)	24/89 (27.0%)	50/239 (20.9%)

Interestingly, *pks*+ *E. coli* strains persistently colonized 10 individual monkeys during the 5-year survey, while persistent colonization by *cnf1*+ *E. coli* was observed in seven macaques. There were two *E. coli* strains isolated from two rhesus; one from a cephalic recording chamber and one from the implant-margin skin site. These two β-hemolytic isolates belonged to the B2 group, were *pks*+/*cnf1*+, and were identified as API code 5144572.

Close correlation was observed between the PCR results and microbiological characteristics (β-hemolysis and API code). Almost all (94.0%) β-hemolytic isolates harbored the *cnf1* gene. API code 1144572 was related to *pks*−/*cnf1*+ isolates, whereas *pks*+/*cnf1*− and *pks*+/*cnf1*+ isolates were associated with API codes 5144572 and 5144552.

### Phylogenetic distribution of *E. coli* strains isolated from macaques

**Fig. 2:**
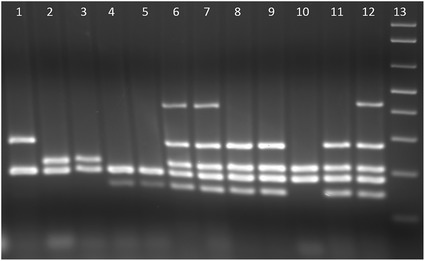
Phylogenetic group determination of *E. coli* isolates from macaques. Lane 1, group D macaque* E. coli*; lanes 2 and 3, group A macaque *E. coli*; lanes 4 and 5, group B1 macaque *E. coli*; lanes 6 and 7, group B2_1_ macaque *E. coli*; lanes 8 and 9, group B2 macaque *E. coli*; lane 10, group A control *E. coli* K12; lane 11, group B2 control *E. coli *NC101; lane 12, group B2_1_ control *E. coli* RS218 (from human meningitis); lane 13, 1 kb+ molecular marker

**Fig. 3:**
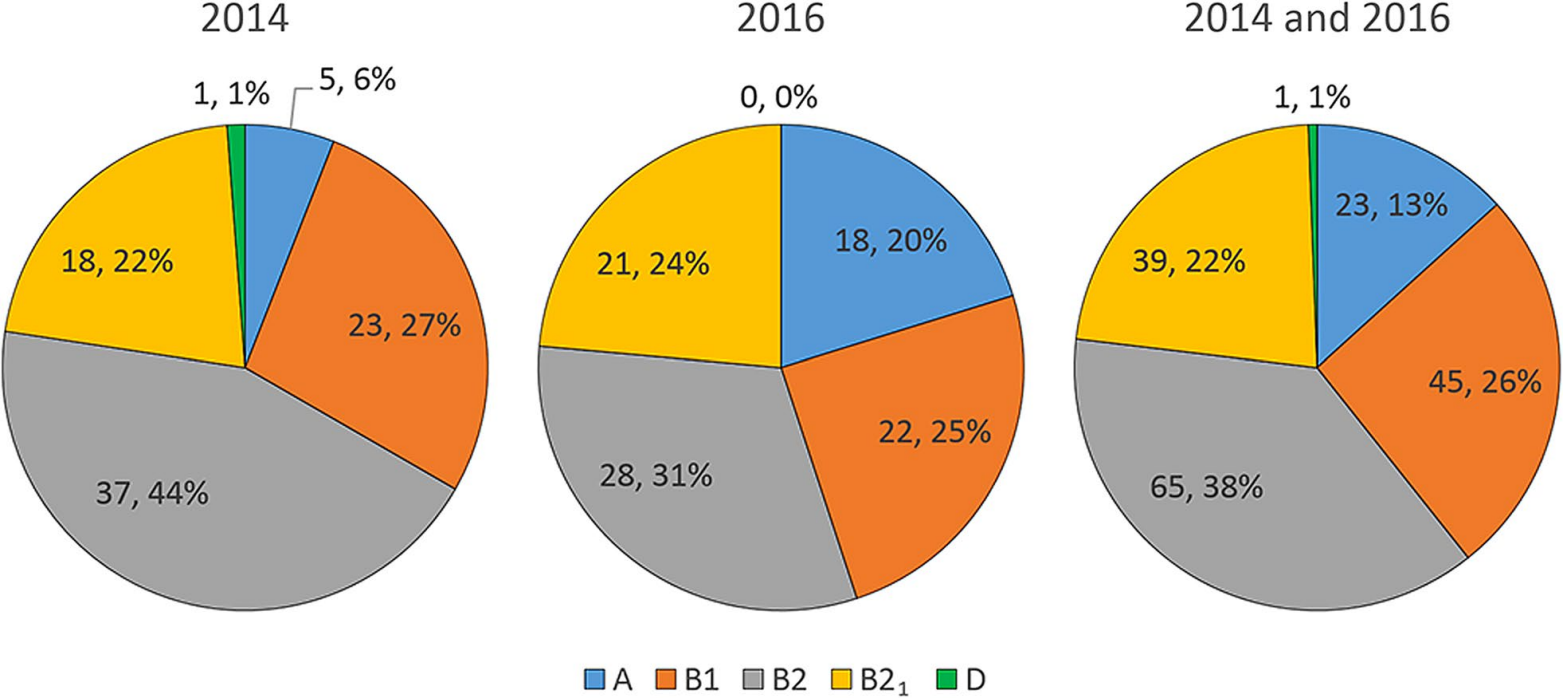
The phylogenetic distribution of *E. coli* isolates from macaques in 2014 and 2016

Based on the PCR amplification pattern of multiplex PCR amplifying five genes (*yjaA, TspE4.C2, chuA, svg*, and *uidA*), the *E. coli* phylogenetic groups were defined as A, B1, B2, B2_1_, and D groups (Fig. 2). The frequency of these groups in *E. coli* isolates from this study (2014 and 2016) is shown in Table 3 and Fig. 3. The frequency distribution of the phylogenetic groups among the *E. coli* isolates was comparable between 2014 and 2016. Based on the total from 2014 to 2016 combined, the most common phylogenetic group was the B2 group (37.6%) followed by B1 (26.0%), B2_1_ (22.5%), A (13.3%), and D (0.6%).

**Table 3:** Phylogenetic distribution of *E. coli* isolates from macaques

Phylogenetic group	2014	2016	Total
A	5/84 (6.0%)	18/89 (20.2%)	23/173 (13.3%)
B1	23/84 (27.4%)	22/89 (24.7%)	45/173 (26.0%)
B2	37/84 (44.0%)	28/89 (31.5%)	65/173 (37.6%)
B2_1_	18/84 (21.4%)	21/89 (23.6%)	39/173 (22.5%)
D	1/84 (1.2%)	0/89 (0.0%)	1/173 (0.6%)

**Fig. 4:**
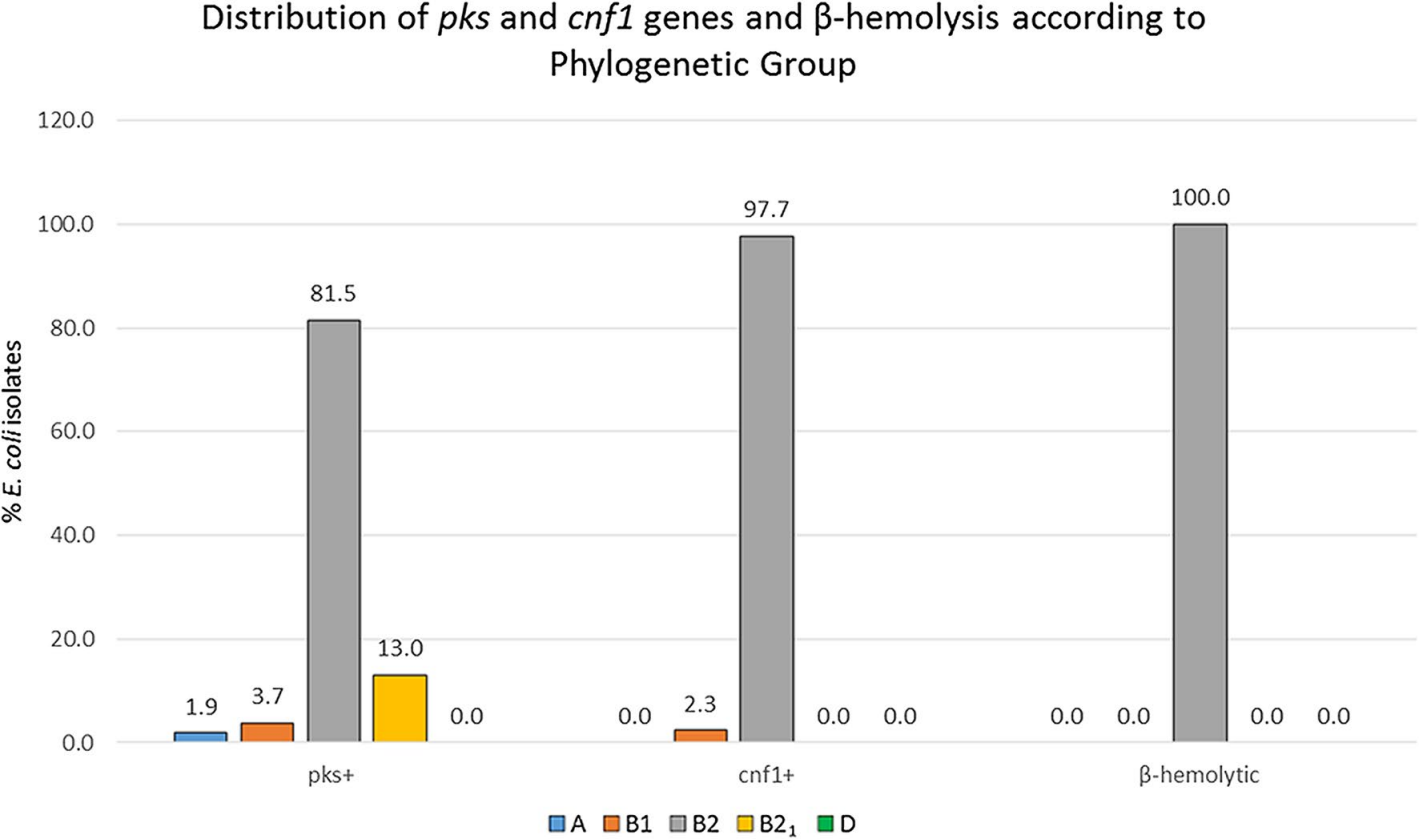
Distribution of *pks* and *cnf1* genes and β-hemolysis according to phylogenetic group of *E. coli* isolates from macaques in 2014 and 2016

The distribution of *pks*, *cnf1*, and β-hemolysis in *E. coli* strains according to phylogenetic groups is illustrated in Table 4 and Fig. 4. All β-hemolytic *E. coli* and all *cnf1*+ *E. coli* (except for one isolate) belonged to the B2 phylogenetic group. *E. coli* strains encoding *pks* genes belonged predominately to the B2 and B2_1_ groups.

**Table 4:** Distribution of *pks*, *cnf1* genes and β-hemolysis in *E. coli* isolates according to phylogenetic group

Phylogenetic group	*pks*+			*cnf1*+			β-hemolytic		
	2014	2016	Total	2014	2016	Total	2014	2016	Total
A	0/36 (0.0%)	1/18 (5.6%)	1/54 (1.9%)	0/19 (0.0%)	0/24 (0.0%)	0/43 (0.0%)	0/16 (0.0%)	0/23 (0.0%)	0/39 (0.0%)
B1	1/36 (2.8%)	1/18 (5.6%)	2/54 (3.7%)	0/19 (0.0%)	1/24 (4.2%)	1/43 (2.3%)	0/16 (0.0%)	0/23 (0.0%)	0/39 (0.0%)
B2	33/36 (91.7%)	11/18 (61.1%)	44/54 (81.5%)	19/19 (100%)	23/24 (95.8%)	42/43 (97.7%)	16/16 (100%)	23/23 (100%)	39/39 (100%)
B2_1_	2/36 (5.6%)	5/18 (27.8%)	7/54 (13.0%)	0/19 (0.0%)	0/24 (0.0%)	0/43 (0.0%)	0/16 (0.0%)	0/23 (0.0%)	0/39 (0.0%)
D	0/36 (0.0%)	0/18 (0.0%)	0/54 (0.0%)	0/19 (0.0%)	0/24 (0.0%)	0/43 (0.0%)	0/16 (0.0%)	0/23 (0.0%)	0/39 (0.0%)

### Serotyping

The serotype for two *pks*+/*cnf1*+ isolates (S1 and S2) was O88:H4, for five *pks*−/*cnf1*+ isolates (S3, S6, S9, S10, and S11) was O25:H4, and for two *pks*+/*cnf1*− isolates (S4 and S5) was O7:H7 (Table 5). The two *pks*−/*cnf1*− isolates (S7 and S8) had two different serotypes: OM:H14 and OM:H16 (Table 5). All five *pks*−/*cnf1*+ isolates (S3, S6, S9, S10, and S11) and both *pks*+/*cnf1*+ isolates (S1 and S2) were confirmed positive for the *cnf1* gene, and none of the isolates serotyped were positive for *elt, estA, estB, stx1, stx2, eae* and *cnf2* genes (Table 5).

**Table 5:** Serotype and virulence factors testing results of *E. coli* isolates from macaques

Sample #	O type	H type	*elt*	*estA*	*estB*	*stx1*	*stx2*	*eae*	*cnf1*	*cnf2*
S1 (*pks*+/*cnf1*+)	88	4	NEG	NEG	NEG	NEG	NEG	NEG	POS	NEG
S2 (*pks*+/*cnf1*+)	88	4	NEG	NEG	NEG	NEG	NEG	NEG	POS	NEG
S3 (*pks*−/*cnf1*+)	25	4	NEG	NEG	NEG	NEG	NEG	NEG	POS	NEG
S6 (*pks*−/*cnf1*+)	25	4	NEG	NEG	NEG	NEG	NEG	NEG	POS	NEG
S9 (*pks*−/*cnf1*+)	25	4	NEG	NEG	NEG	NEG	NEG	NEG	POS	NEG
S10 (*pks*−/*cnf1*+)	25	4	NEG	NEG	NEG	NEG	NEG	NEG	POS	NEG
S11 (*pks*−/*cnf1*+)	25	4	NEG	NEG	NEG	NEG	NEG	NEG	POS	NEG
S4 (*pks*+/*cnf1*−)	7	7	NEG	NEG	NEG	NEG	NEG	NEG	NEG	NEG
S5 (*pks*+/*cnf1*−)	7	7	NEG	NEG	NEG	NEG	NEG	NEG	NEG	NEG
S7 (*pks*−/*cnf1*−)	M	14	NEG	NEG	NEG	NEG	NEG	NEG	NEG	NEG
S8 (*pks*−/*cnf1*−)	M	16	NEG	NEG	NEG	NEG	NEG	NEG	NEG	NEG

### In vitro cytotoxicity of *E. coli* isolates from macaques

Previous studies have found that the cyclomodulins colibactin, CNF, CDT, and CIF cause megalocytic-like cytotoxicity to cell lines in vitro; however this effect by colibactin and CIF is dependent on contact with live bacteria, while for CNF and CDT is observable only with bacterial sonicate or supernatant treatment [11]. Therefore, we tested representative *E. coli* isolates using cell culture assays to confirm colibactin and CNF1 cytotoxic activity predicted by the PCR results.

**Fig. 5:**
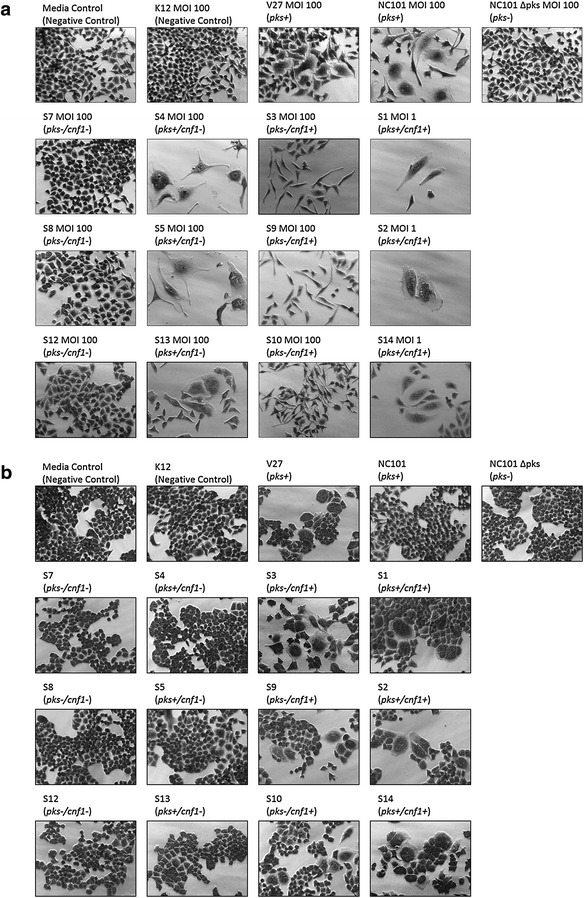
**a** Cell culture assay for cytotoxicity. HeLa cells were treated with *E*. *coli* at a multiplicity of infection (MOI) between 1 and 100 for 4 h followed by a 72 h incubation in gentamicin-containing media. Cells infected with the novel rhesus macaque isolates encoding *pks* (S1, S2, S4, S5, S13, and S14) displayed megalocytosis (enlargement of the cell body and nucleus) similar to the *pks*+ *E. coli* controls (NC101 mouse isolate and V27 human urosepsis isolate). Isolates *pks*−/*cnf1*+ (S3, S9, and S10) did not induce megalocytosis, but caused elongated cell morphologies. No cytotoxicity was observed for cells treated with novel isolates *pks*−/*cnf1*− (S7, S8, and S12), the *E. coli* negative controls (media control and K12 non-pathogenic laboratory strain) as well as NC101 Δ*pks*. Images were taken at 20× magnification. **b** Cell culture assay for cytotoxicity. HeLa cells were treated for 72 h with *E. coli* sonicate at a dose of 40 μg/mL total protein. Only treatment with sonicate from the *cnf1*-encoding novel rhesus macaque isolates (S1, S2, S3, S9, S10, and S14) caused cell body enlargement and multi-nucleation. No cytotoxicity was observed after sonicate treatment with the other novel isolates. Images were taken at 20× magnification

Transient infection with *pks*+/*cnf1*− *E. coli* isolates (S4, S5, and S13) at MOI 100 caused contact-dependent megalocytosis (Fig. 5a); however, sonicate treatment with these isolates did not cause observable cytotoxicity (Fig. 5b). Infection with *pks*+/*cnf1*+ *E. coli* isolates (S1, S2, and S14) killed all HeLa cells at MOI ≥ 5. Surviving HeLa cells at MOI 1 appeared megalocytic (Fig. 5a). Sonicate treatment by these isolates also caused HeLa cell body distention and multi-nucleation (Fig. 5b). *E. coli* isolates that were *pks*−/*cnf1*+ (S3, S9, and S10) did not cause megalocytosis to HeLa cells after infection, but instead caused a cytotoxic effect observed as cellular elongation (Fig. 5a). Sonicate treatment with these isolates caused HeLa cells to become distended with multiple nuclei (Fig. 5b). HeLa cells treated with live bacteria or sonicate from *pks*−/*cnf1*− (S7, S8, and S12) appeared indistinguishable from those treated with media, K12, and NC101 Δpks negative controls (Fig. 5). Megalocytic CNF1 cytotoxicity was also observed only when HeLa cells were treated with culture supernatant from the *cnf1*+ isolates (S3 and S14) (Additional file 2: Figure S1). All isolates were found by PCR to be negative for *cdt* and *cif* genes, therefore excluding the influence of these cyclomodulins. Together, these results demonstrate that the novel *E. coli* isolates exhibit colibactin and CNF1 cytotoxicity, as predicted by their genotype.

### Draft genome sequencing and comparative analysis

The draft genomes of ten representative rhesus macaque *E. coli* isolates were sequenced for identification of virulence factor and antimicrobial resistance genes, as well as for comparative analysis. Genome sizes, GC content, and the number of annotated protein coding and RNA genes were comparable between the different rhesus macaque *E. coli* isolates and representative pathogenic and non-pathogenic *E. coli* strains (Table 6).

**Table 6:** Genome characteristics

Strain/isolate	Source	Genome length (bp)	Contigs	N50	GC content (%)	Protein genes (CDS)	tRNA genes	rRNA genes	Virulence factor genes	Antibiotic resistance genes	GenBank accession
S1 (*pks*+/*cnf1*+)	Research rhesus macaque rectal swab	5,070,329	92	268,704	50.49	5022	78	10	*astA, celb, cnf1, gad, hlyCABD, iroN, iss, mchB, mchC, mchF, mcmA, pks, vat*	–	NHZD00000000
S2 (*pks*+/*cnf1*+)	Research rhesus macaque rectal swab	5,070,134	98	268,677	50.49	5017	78	10	*astA, celb, cnf1, gad, hlyCABD, iroN, iss, mchB, mchC, mchF, mcmA, pks, vat*	–	NHZC00000000
S4 (*pks*+/*cnf1*−)	Research rhesus macaque rectal swab	4,843,133	160	65,852	50.72	4721	83	10	*celb, gad, iroN, iss, mchB, mchC, mchF, mcmA, pic, pks, vat*	–	NHZA00000000
S5 (*pks*+/*cnf1*−)	Research rhesus macaque rectal swab	4,899,189	116	127,782	50.54	4791	81	10	*celb, gad, iroN, iss, mchB, mchC, mchF, mcmA, pic, pks, vat*	–	NHYZ00000000
S3 (*pks*−/*cnf1*+)	Research rhesus macaque rectal swab	5,239,168	227	105,395	50.66	5403	80	8	*cnf1, hlyCABD, gad, iha, iss, sat*	*aac(6*′*)Ib*-*cr, blaCTX*-*M*-*15, blaOXA*-*1, catB3*-*like, tet(A)*	NHZB00000000
S6 (*pks*−/*cnf1*+)	Research rhesus macaque rectal swab	5,345,115	176	178,747	50.53	5502	79	9	*cnf1, hlyCABD, gad, iha, iss, sat*	*aac(6*′*)Ib*-*cr, blaCTX*-*M*-*15, blaOXA*-*1, catB3*-*like, tet(A)*	NHYY00000000
S9 (*pks*−/*cnf1*+)	Research rhesus macaque rectal swab	5,536,267	173	170,757	50.32	5765	81	9	*cnf1, hlyCABD, gad, iha, iss, sat*	*aac(6*′*)Ib*-*cr, blaCTX*-*M*-*15, blaOXA*-*1, catB3*-*like, tet(A)*	NHYV00000000
S10 (*pks*−/*cnf1*+)	Research rhesus macaque rectal swab	5,254,454	178	113,006	50.70	5393	80	9	*cnf1, hlyCABD, gad, iha, iss, sat*	*aac(6*′*)Ib*-*cr, blaCTX*-*M*-*15, blaOXA*-*1, catB3*-*like, tet(A)*	NHYU00000000
S7 (*pks*−/*cnf1*−)	Research rhesus macaque rectal swab	4,840,594	136	147,699	50.76	4807	84	10	*gad, iss, lpfA*	–	NHYX00000000
S8 (*pks*−/*cnf1*−)	Research rhesus macaque rectal swab	4,770,474	119	168,371	50.68	4730	81	11	*gad, iss, lpfA*	–	NHYW00000000
IHE3034	Human neonatal meningitis	5,108,383	1 (complete genome)	–	50.70	5045	97	22	*gad, iroN, iss, pks, sfaS, vat*	–	CP001969.1
NC101	Research mouse intestinal commensal, pro-carcinogenic	5,021,144	27	511,891	50.57	4917	72	4	*gad, iroN, iss, pks, sfaS, vat*	–	AEFA00000000.1
UTI89	Human uropathogenic strain	5,179,971	1 (complete genome)	–	50.60	5040	89	14	*cnf1, gad, gad, iroN, iss, senB, sfaS, vat*	–	CP000243.1
K-12 substr. DH10B	Human non-pathogenic intestinal commensal	4,686,137	1 (complete genome)	–	50.80	4606	87	14	*gad, iss*	–	AP012306.1

*Escherichia coli* isolates that were PCR-positive for *pks* and/or *cnf1* genes also had the full-length gene sequences detected in their annotated genomes. Additionally, sequences for the cyclomodulins *cnf2*, *cdt*, and *cif*, as well as the virulence factor genes *elt*, *estA*, *estB*, *stx1*, *stx2*, and *eae* were not detected in any of the genomes, which agrees with previous PCR and serotyping results.

**Fig. 6:**
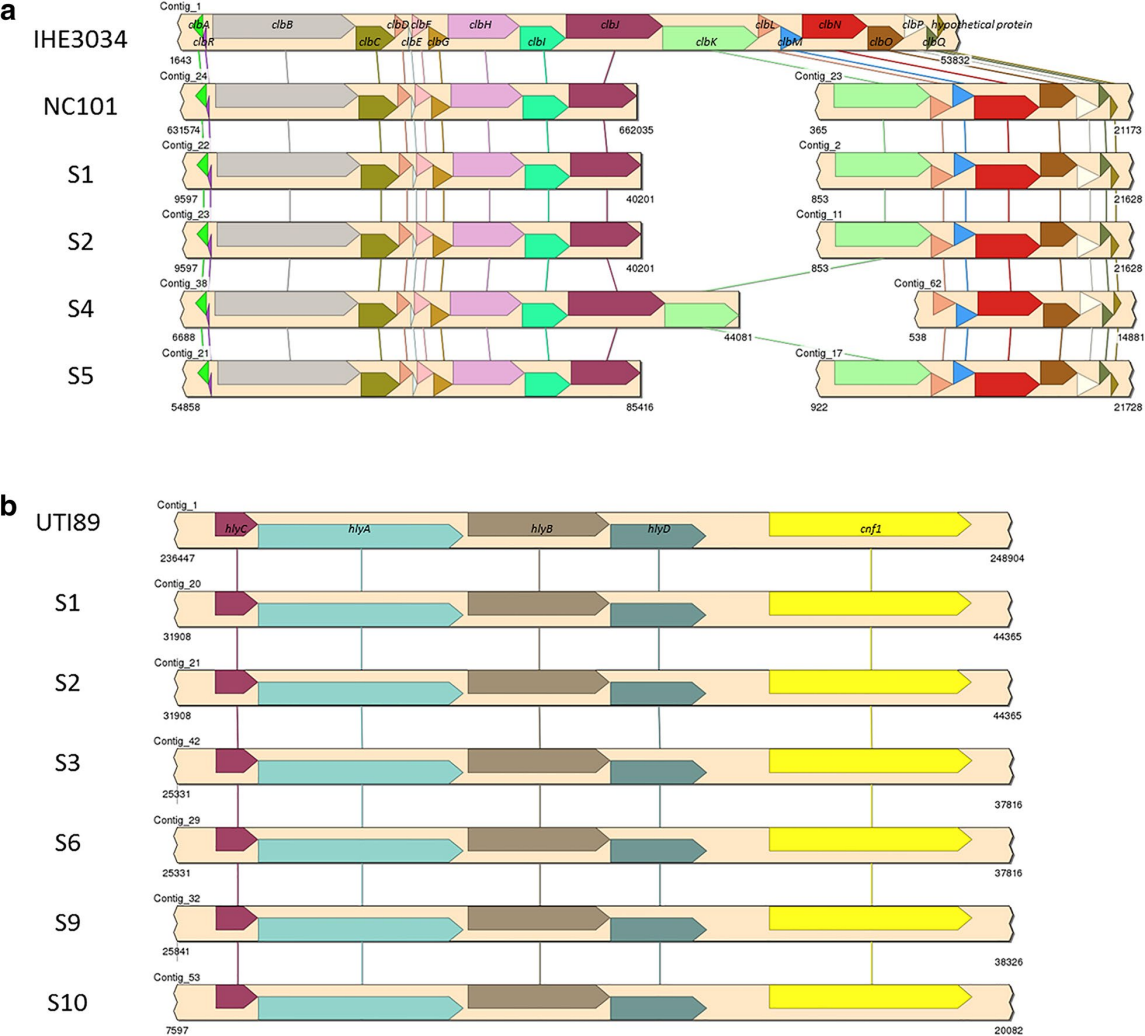
**a** In the draft genomes of four representative novel rhesus macaque isolates PCR-positive for the *clbA* and *clbQ* genes, complete *pks* pathogenicity islands were identified and had ≥ 98% sequence homology and identical syntenic relationships compared to prototype *pks*+ strains IHE3034 and NC101. Due to being draft genomes, the genes in *pks* pathogenicity islands were separated onto two different contigs for NC101 and the four novel isolates. No *pks* pathogenicity island genes were identified in the draft genomes of any of the *pks*− isolates. **b** In the draft genomes of six representative novel rhesus macaque isolates PCR-positive for *cnf1*, the hemolysin-*cnf1* operon was identified and had ≥ 99% sequence homology and identical syntenic relationships compared to the prototype hemolysin-*cnf1* operon of the human uropathogenic *E. coli* strain UTI89. Neither *cnf1* nor any hemolysin genes belonging to this operon were detected in any of the draft genomes of the other representative novel isolates

Complete *pks* gene islands were identified in isolates S1, S2, S4, and S5. All *pks* island genes showed ≥ 98% sequence homology and identical syntenic relationships compared to prototype *pks*+ strains IHE3034 and NC101 (Fig. 6a). Likewise, all isolates positive for *cnf1* had the β-hemolysin genes *hlyCABD* immediately upstream, consistent with the hemolysin-*cnf1* operon found in uropathogenic *E. coli* (UPEC) such as UTI89 (Fig. 6b).

Other putative virulence factor genes were also detected in the *E. coli* isolates (Table 6). These virulence factor genes represent toxins (*astA, pic, vat, sat*), survival and immune evasion factors (*gad, iss*), iron acquisition (*iroN*), adherence (*iha*, *lpfA*) and bacteriocin synthesis (*celb*, *mchB*, *mchC*, *mchF*, *mcmA*). These virulence genes are associated with host colonization and pathogenicity in intestinal and extra-intestinal diseases (Additional file 1: Table S2).

## Discussion

*Escherichia coli* is a normal inhabitant of the gastrointestinal tract of macaques and several different serotypes of bacteria have been isolated from asymptomatic rhesus macaques [32]. To our knowledge this is the first reported isolation of colibactin-encoding *E. coli* strains from macaques. Colibactin was first identified in several *E. coli* strains by Oswald and co-workers in 2006 [12]. Colibactin, encoded by a 54 kb gene cluster (the *pks* island), is a genotoxin which causes DNA double strand breaks and activation of the DNA damage checkpoint pathway, leading to cell cycle arrest and eventually cell death. The role of colibactin-encoding *E. coli* has been explored in human colorectal cancer and investigated in different types of mouse models including IL10^−/−^ mice treated with azoxymethane (AOM), C57BL/6J-ApcMin/J mice treated with AOM and dextran sodium sulfate (DSS), and nude mice with xenografts [11, 15, 33, 34]. These studies have established a role of *pks*-encoding *E. coli* in inflammation and cancer. The increase in *E. coli* growth and colonization may be due to inflammation-generated nitrate, as *E. coli*, a facultative anaerobe, can produce energy through the use of nitrate, *S*-oxides and *N*-oxides as terminal electron acceptors for anaerobic respiration and thus outcompete obligate anaerobes, the major colonizers of the lower bowel [35].

Among the *E. coli* isolates from macaques, 30.1% of isolates were *pks*+ strains and colibactin activities were confirmed in selected isolates. The prevalence of *pks*+ *E. coli* colonization in macaques was similar to the 25% prevalence of *pks*+ B2 *E. coli* colonization in humans [36]. Several publications have revealed the higher prevalence of *pks*+ *E. coli* strains in biopsies from colorectal cancer (CRC) patients (66.7% in CRC patients, 40% in IBD patients and 20.8% in no IBD/no CRC controls) [11, 15, 37].

The 20.9% prevalence of *cnf1*+ *E. coli* in this study was consistent with our previous findings [21]. Close correlation was observed between the *cnf1* gene and β-hemolysis in *E. coli* isolates. This association was reported in our previous studies [20, 21] and by other authors [19, 38]. In the *E. coli* J96 strain and other strains, the *cnf1* gene is located downstream of hemolysin (*hlyCABD*) in pathogenicity island II (PAI II), and the expression of *cnf1* is regulated by the hemolysin promoter [39–41]. Likewise, our draft genome sequences of macaque *E. coli* strains indicate that hemolysin (*hlyCABD*) is located directly upstream of the *cnf1* gene. These two toxins could therefore be associated with enhanced virulence. In our in vitro cytotoxicity assays, infection with the *pks*−/*cnf1*+ isolates (S3, S9, S10) caused ~ 50% of HeLa cells to die, and the surviving cells exhibited an elongated morphology; CNF1 cytotoxicity by these isolates was only observed with sonicate and supernatant treatment. The draft genome sequence data showed that only *pks*−/*cnf1*+ isolates (S3, S6, S9, and S10) also contained an annotated secreted autotransporter toxin (*sat*), which is expressed by some UPEC strains and reported to cause cell elongation in vitro [42]. We speculate that expression of *sat* by these isolates may have caused HeLa cells to adopt an elongated morphology. Interestingly, we also identified *E. coli* strains co-harboring the *pks* and *cnf1* genes. Thirty-one (13.0%) *E. coli* isolates were positive for both *pks* and *cnf1* (*pks*+/*cnf1*+). The *E. coli* strains co-harboring these two toxins have also been reported in human samples [10, 11]. The authors noted that 15% of *E. coli* isolates from urosepsis patients and from the feces of healthy individuals were double-positive for *pks* and *cnf1* (*pks*+/*cnf1*+). In our in vitro cytotoxicity assays, the isolates *pks*+/*cnf1*+ (S1, S2, and S14) exhibited severe toxicity to HeLa cells, given all cells were dead when treated with these live isolates at MOI 5, 25 and 100 (Fig. 5).

Yasuda and co-workers characterized biogeographic relationships in the rhesus macaque intestinal microbiome and found that stool microbiota was highly representative of the colonic lumen and mucosa, which were respectively enriched in obligate and facultative anaerobes [43]. In our study the *E. coli* strains were isolated from rectal swab samples and may, by analogy, be representative of the *E. coli* strains colonized in the macaque gastrointestinal tract. Moreover, among *E. coli* isolates in the present study, two isolates which were β-hemolytic and *pks*+/*cnf1*+ were isolated from a cephalic recording chamber and implant-margin skin of two macaques. This raises the concern that these *E. coli* isolates may cause meningitis in macaques used in neurobiological research based on the previous reports that *pks*+ *E. coli* were isolated and related to meningitis in humans and animals [44].

For the *E. coli* isolates from macaques in this study, the predominant phylogenetic group was B2 group including B2_1_ subgroup (60.1%) followed by B1 (26.0%), A (13.3%), and D group (0.6%). The distribution of phylogenetic groups of macaque *E. coli* strains were similar to distribution of *E. coli* isolates from healthy humans [10, 11]. The distribution of cyclomodulin-encoding genes (*pks, cnf*, *cdt* and *cif*) in relation to the phylogenetic background in *E. coli* isolates from urosepsis patients and healthy individuals indicated that strains *pks*+ and/or *cnf* *1*+ strongly associated with the B2 group [10]. In another study, the prevalence of *E. coli* producing cyclomodulins and genotoxins in colon cancer had a higher prevalence of the B2 phylogenetic group *E. coli* harboring the *pks* gene (55.0%) and *cnf1* gene (39.5%) in biopsies of patients with colorectal cancer than that in patients with diverticulosis (19.3% *pks*+ and 12.9% *cnf1*+) [11]. In both studies, the percentage of *E. coli* strains harboring *cdt* and *cif* genes were much lower (1–6%). Representative isolates in our study were negative for *cdt* and *cif* according to PCR, in vitro cytotoxicity assay, and genome analysis results. We found that *pks*+, *cnf1*+, and β-hemolytic *E. coli* strains belonged to group B2. 49.0% (51 out of 104) of the isolates belonging to B2 (including the B2_1_ subgroup) were *pks*+, 40.4% (42 out of 104) were *cnf1*+, and 37.5% (39 out of 104) were β-hemolytic. Seven *pks*+ *E. coli* isolates belonged to the B2_1_ subgroup, which is a highly virulent phylogenetic subgroup among extra-intestinal pathogenic *E. coli* B2 strains [7, 44].

The serotype data in the present study revealed that the serotype of selected *E. coli* strains corresponded to their toxin-harboring content. Of the isolates serotyped, those *pks*+/*cnf1*+ (S1 and S2) were the O88:H4 serotype. The *pks*−/*cnf1*+ isolates (S3, S6, S9, S10, S11) were O25:H4, the *pks*+/*cnf1*− isolates (S4 and S5) were O7:H7, the *pks*−/*cnf1*− isolates (S7 and S8) were OM:H14 or OM:H16. The O7:H7:K1 serotype belonging to phylogenetic group B2 was cultured from IL10^−/−^ and wild-type mice [45]. In these experiments, cecal and colonic inflammation observed in IL10^−/−^ mice was accompanied by diminished intestinal microbial diversity and a higher number of *E. coli* organisms compared to wild-type mice [45]. Serotype O7:H7 *E. coli* strains were also found among Shiga-toxin-producing *E. coli* strain isolated from calves in Brazil [46]. UPEC O25:H4 strains were reported in patients with urinary tract infection [47] and patients with cystitis or prostatitis [48]. These strains belonging to group B2 had multiple antibiotic drug resistance. By analyzing the draft genome sequences of our macaque *E. coli* isolates, putative multi-antibiotic resistance genes were identified exclusively in *cnf1*+ strains (serotype O25:H4). These included resistance genes to the tetracycline (*tet(A)*), phenicol (*catB3*-*like*), fluoroquinolone and aminoglycoside (*aac(6′6′)Ib*-*cr*), and β-lactam (*blaCTX*-*M*-*15*, *blaOXA*-*1*) classes of antibiotics, consistent with our previous antibiotic resistance findings of *cnf1*+ *E. coli* strains in macaques [21]. Other studies have noted that antibiotic-susceptibility is inversely related to the number of virulence factor genes present in extra-intestinal *E. coli* strains [49]. Similarly, in this study isolates that were *pks*−/*cnf1*+ had the second fewest number of virulence factor genes and also were the only isolates with putative antibiotic resistance genes detected.

Different virulence factor gene profiles appear to be present depending on whether the isolates were *pks*+/*cnf1*+, *pks*+/*cnf1*−, *pks*−/*cnf1*+, or *pks*−/*cnf1*−. The number and prolife of these virulence genes agrees with in vitro cytotoxicity to HeLa cells in that the isolates showing pronounced cytotoxicity (*pks*+ and/or *cnf1*+ strains) had substantially more virulence factor genes present in their genomes compared to the less cytotoxic *pks*−/*cnf1*− isolates. Except for *lpfA*, the *pks*−/*cnf1*− *E. coli* isolates had the same virulence factor genes as K12, suggesting that along with the in vitro cytotoxicity results, these isolates likely have attenuated pathogenic potential. Interestingly, *pks*+ strains harboring the bacteriocin synthesis genes for colicin E2 and microcin H47 also had the most virulence genes. This agrees with other studies reporting that *E. coli* strains expressing bacteriocins are statistically more likely to co-associate with more virulence factor genes in their genomes compared to strains that lack bacteriocin potential [49–51]. Likewise, bacteriocin genes are found more frequently in *E. coli* strains belonging to the pathogenic B2 or D phylogroup, such as our monkey isolates [49, 50, 52]. In particular, microcin H47 is predominantly found in the UPEC strains [53]. It is hypothesized that bacteriocin activity by pathogenic *E. coli* may provide a competitive survival and colonization advantage against commensal organisms, especially when availability of essential nutrients, like iron, is scarce [51].

In summary, *E. coli* strains encoding colibactin, CNF1, or both were identified in macaques. The *pks*+ and/or *cnf1*+ isolates belonged to phylogenetic group B2 and induced cytotoxic effects to HeLa cells in vitro. The genomic data supports the presence of virulence factor and antibiotic resistance genes in these isolates and suggests that they may have the pathogenic potential to influence clinical and subclinical disease. The impact of these strains on the health of macaques is unclear as analysis of medical records did not allow an association of clinical events and isolation of *E. coli*. Given colibactin and CNF1-encoding *E. coli* has been isolated from human and animals populations, there is a concern about potential zoonotic spread. The presence of colibactin and CNF1-producing *E. coli* strains in primates used in neurobiology emphasizes the importance of appropriate personnel protection and hygiene practices when handling these primates.

## Conclusions

The prevalence of *pks*+ *E. coli* in rhesus macaques is not known, nor is there published evidence that *E. coli* strains encoding both *pks* and *cnf1* genes colonize macaques. In the present study, *E. coli* strains encoding colibactin and CNF1 were identified in the rectal swabs and extra-intestinal samples of macaques sampled over a 3-year period. Among the 239 isolates, 72 (30.1%) were positive for *pks* genes and 50 (20.9%) were *cnf1*+. Our findings indicate that colibactin and CNF1-encoding *E. coli* colonizing laboratory macaques can potentially cause clinical and subclinical diseases that impact studies conducted in macaque models.

## Additional files

**Additional file 1: Table S1.** Genes, primers, and annealing temperature used for amplification. **Table S2.** Virulence genes.

**Additional file 2: Figure S1.** Only treatment with supernatant from the *cnf1*-encoding novel rhesus macaque isolates (S3, S14) caused cell body enlargement and multi-nucleation. No cytotoxicity was observed after supernatant treatment with the other novel isolates. Images were taken at 20× magnification.

## References

[1] Leser TD, Molbak L (2009). Better living through microbial action: the benefits of the mammalian gastrointestinal microbiota on the host. Environ Microbiol.

[2] Kaper JB, Nataro JP, Mobley HL (2004). Pathogenic *Escherichia coli*. Nat Rev Microbiol.

[3] Croxen MA, Finlay BB (2010). Molecular mechanisms of *Escherichia coli* pathogenicity. Nat Rev Microbiol.

[4] Clermont O, Bonacorsi S, Bingen E (2000). Rapid and simple determination of the *Escherichia coli* phylogenetic group. Appl Environ Microbiol.

[5] Herzer PJ (1990). Phylogenetic distribution of branched RNA-linked multicopy single-stranded DNA among natural isolates of *Escherichia coli*. J Bacteriol.

[6] Carlos C (2010). *Escherichia coli* phylogenetic group determination and its application in the identification of the major animal source of fecal contamination. BMC Microbiol.

[7] Bidet P (2007). Detection and identification by PCR of a highly virulent phylogenetic subgroup among extraintestinal pathogenic *Escherichia coli* B2 strains. Appl Environ Microbiol.

[8] Picard B (1999). The link between phylogeny and virulence in *Escherichia coli* extraintestinal infection. Infect Immun.

[9] Escobar-Paramo P (2006). Identification of forces shaping the commensal *Escherichia coli* genetic structure by comparing animal and human isolates. Environ Microbiol.

[10] Dubois D (2010). Cyclomodulins in urosepsis strains of *Escherichia coli*. J Clin Microbiol.

[11] Buc E (2013). High prevalence of mucosa-associated *E. coli* producing cyclomodulin and genotoxin in colon cancer. PLoS ONE.

[12] Nougayrede JP (2006). *Escherichia coli* induces DNA double-strand breaks in eukaryotic cells. Science.

[13] Putze J (2009). Genetic structure and distribution of the colibactin genomic island among members of the family Enterobacteriaceae. Infect Immun.

[14] Kim SC (2005). Variable phenotypes of enterocolitis in interleukin 10-deficient mice monoassociated with two different commensal bacteria. Gastroenterology.

[15] Arthur JC (2012). Intestinal inflammation targets cancer-inducing activity of the microbiota. Science.

[16] Garcia A (2016). Cytotoxic *Escherichia coli* strains encoding colibactin colonize laboratory mice. Microbes Infect.

[17] Falbo V (1993). Isolation and nucleotide sequence of the gene encoding cytotoxic necrotizing factor 1 of *Escherichia coli*. Infect Immun.

[18] Oswald E (1989). Cytotoxic effect of multinucleation in HeLa cell cultures associated with the presence of Vir plasmid in *Escherichia coli* strains. FEMS Microbiol Lett.

[19] Landraud L (2000). Frequency of *Escherichia coli* strains producing the cytotoxic necrotizing factor (CNF1) in nosocomial urinary tract infections. Lett Appl Microbiol.

[20] Marini RP (2004). Characterization of hemolytic *Escherichia coli* strains in ferrets: recognition of candidate virulence factor CNF1. J Clin Microbiol.

[21] Martin HR (2009). Characterization of cytotoxic necrotizing factor 1-producing *Escherichia coli* strains from faeces of healthy macaques. J Med Microbiol.

[22] Feria C (2001). Virulence genes and P fimbriae PapA subunit diversity in canine and feline uropathogenic *Escherichia coli*. Vet Microbiol.

[23] Johnson JR (2003). Identification of urovirulence traits in *Escherichia coli* by comparison of urinary and rectal *E. coli* isolates from dogs with urinary tract infection. J Clin Microbiol.

[24] Siqueira AK (2009). Virulence factors in *Escherichia coli* strains isolated from urinary tract infection and pyometra cases and from feces of healthy dogs. Res Vet Sci.

[25] Toth I (2000). Characterization of intestinal *cnf1*+ *Escherichia coli* from weaned pigs. Int J Med Microbiol.

[26] Rodriguez-Siek KE (2005). Comparison of *Escherichia coli* isolates implicated in human urinary tract infection and avian colibacillosis. Microbiology.

[27] Wattam AR (2017). Improvements to PATRIC, the all-bacterial bioinformatics database and analysis resource center. Nucleic Acids Res.

[28] Joensen KG (2014). Real-time whole-genome sequencing for routine typing, surveillance, and outbreak detection of verotoxigenic *Escherichia coli*. J Clin Microbiol.

[29] Zankari E (2012). Identification of acquired antimicrobial resistance genes. J Antimicrob Chemother.

[30] Veltri D, Wight MM, Crouch JA (2016). SimpleSynteny: a web-based tool for visualization of microsynteny across multiple species. Nucleic Acids Res.

[31] Frydman GH (2017). Local and systemic changes associated with long-term, percutaneous, static implantation of titanium alloys in rhesus macaques (*Macaca mulatta*). Comp Med.

[32] Schiff LJ (1972). Enteropathogenic *Escherichia coli* infections: increasing awareness of a problem in laboratory animals. Lab Anim Sci.

[33] Cougnoux A (2014). Bacterial genotoxin colibactin promotes colon tumour growth by inducing a senescence-associated secretory phenotype. Gut.

[34] Bonnet M (2014). Colonization of the human gut by *E. coli* and colorectal cancer risk. Clin Cancer Res.

[35] Winter SE (2013). Host-derived nitrate boosts growth of *E. coli* in the inflamed gut. Science.

[36] Secher T, Brehin C, Oswald E (2016). Early settlers: which *E. coli* strains do you not want at birth?. Am J Physiol Gastrointest Liver Physiol.

[37] Raisch J (2014). Colon cancer-associated B2 *Escherichia coli* colonize gut mucosa and promote cell proliferation. World J Gastroenterol.

[38] Blanco M (1996). Polymerase chain reaction for detection of *Escherichia coli* strains producing cytotoxic necrotizing factor type 1 and type 2 (CNF1 and CNF2). J Microbiol Methods.

[39] Blum G (1995). Gene clusters encoding the cytotoxic necrotizing factor type 1, Prs-fimbriae and alpha-hemolysin form the pathogenicity island II of the uropathogenic *Escherichia coli* strain J96. FEMS Microbiol Lett.

[40] Landraud L (2003). Expression of cnf1 by *Escherichia coli* J96 involves a large upstream DNA region including the hlyCABD operon, and is regulated by the RfaH protein. Mol Microbiol.

[41] Lemonnier M, Landraud L, Lemichez E (2007). Rho GTPase-activating bacterial toxins: from bacterial virulence regulation to eukaryotic cell biology. FEMS Microbiol Rev.

[42] Guyer DM (2000). Identification of sat, an autotransporter toxin produced by uropathogenic *Escherichia coli*. Mol Microbiol.

[43] Yasuda K (2015). Biogeography of the intestinal mucosal and lumenal microbiome in the rhesus macaque. Cell Host Microbe.

[44] Bonacorsi S (2003). Molecular analysis and experimental virulence of French and North American *Escherichia coli* neonatal meningitis isolates: identification of a new virulent clone. J Infect Dis.

[45] Wohlgemuth S (2009). Reduced microbial diversity and high numbers of one single *Escherichia coli* strain in the intestine of colitic mice. Environ Microbiol.

[46] Aidar-Ugrinovich L (2007). Serotypes, virulence genes, and intimin types of Shiga toxin-producing *Escherichia coli* (STEC) and enteropathogenic *E. coli* (EPEC) isolated from calves in Sao Paulo, Brazil. Int J Food Microbiol.

[47] Molina-Lopez J (2011). Drug resistance, serotypes, and phylogenetic groups among uropathogenic *Escherichia coli* including O25-ST131 in Mexico City. J Infect Dev Ctries.

[48] Morales-Espinosa R (2016). UPEC strain characterization isolated from Mexican patients with recurrent urinary infections. J Infect Dev Ctries.

[49] Budic M (2011). *Escherichia coli* bacteriocins: antimicrobial efficacy and prevalence among isolates from patients with bacteraemia. PLoS ONE.

[50] Abraham S (2012). Molecular characterization of *Escherichia coli* strains that cause symptomatic and asymptomatic urinary tract infections. J Clin Microbiol.

[51] Micenkova L (2014). Bacteriocin-encoding genes and ExPEC virulence determinants are associated in human fecal *Escherichia coli* strains. BMC Microbiol.

[52] Micenkova L (2016). Microcin determinants are associated with B2 phylogroup of human fecal *Escherichia coli* isolates. Microbiologyopen.

[53] Azpiroz MF, Poey ME, Lavina M (2009). Microcins and urovirulence in *Escherichia coli*. Microb Pathog.

