# Supplementary material for: Cytotoxic Escherichia coli strains encoding colibactin and cytotoxic necrotizing factor (CNF) colonize laboratory macaques
Source: Gut Pathog. 2017 Dec 6;9:71. doi: 10.1186/s13099-017-0220-y (PMC5718112; doi:10.1186/s13099-017-0220-y)
Supplement: Supplementary file 1 — Additional file 1: Table S1. Genes, primers, and annealing temperature used for amplification. Table S2. Virulence genes. [file 13099_2017_220_MOESM1_ESM.pdf]

Table S1. Genes, primers, and annealing temperature used for amplification

Gene	Primer designation	Primer sequence (5′ to 3′)	Annealing temperature (°C)	Reference
16S rRNA[a]	9F (F24)	GAGTTTGATYMTGGCTCAG	58	Harper CG,2003
	F16	TAGATACCCYGGTAGTCC		Shen Z,2001
*clb*A[a]	IHAPJPN42 (clbAr)	CAGATACACAGATACCATTCA	55	Johnson JR, 2008, Bubois D, 2010
	IHAPJPN46 (clbAf)	CTAGATTATCCGTGGCGATTC		
*clb*Q[a]	IHAPJPN55 (clbQr)	TTATCCTGTTAGCTTTCGTTC	55	Johnson JR, 2008, Bubois D, 2010
	IHAPJPN56 (clbQf)	CTTGTATAGTTACACAACTATTTC		
Multiplex *cnf*	CNF-s	TTATATAGACAAGATGGA	55	Toth I, 2003
	CNF-as	CATAAAGCTTTACAATATTGA		
*cnf*1	CNF-1s	GGGGGAAGTACAGAAGAATTA	55	Toth I, 2003
	CNF-1as	TTGCCGTCCACTCTCACCAGT		
*cnf*2	CNF-2s	TATCATACGGGCAGGAGGAAGCACC	55	Toth I, 2003
	CNF-2as	GTCACAATAGACAATAATTTTCCG		
multiplex *cdt*	cdt-s1	GAAAGTAAATGGAATATAAATGTCCG	55	Toth I, 2003
	cdt-as1	AAATCACCAAGAATCATCCAGTTA		
*yja*A	YjaA.1	TGAAGTGTCAGGAGACGCTG	55	Clermont D,2000
	YjaA.2	ATGGAGAATGCGTTCCTCAAC		
TSPE4.C2	TspE4C2.1	GAGTAATGTCGGGGCATTCA	55	Clermont D,2000
(DNA fragment)	TspE4C2.2	CGCGCCAACAAAGTATTACG		
*chu*A	ChuA.1	GACGAACCAACGGTCAGGAT	55	Clermont D,2000
	ChuA.2	TGCCGCCAGTACCAAAGACA		
svg	svg.1	TCCGGCTGATTACAAACCAAC	55	Bidet P, 2007
	svg.2	CTGCACGAGGTTGTAGTCCTG		
*uid*A	P3	TATGAACTGTGCGTCACAGCC	55	Heininger A, 1999
	P4	CATCAGCACGTTATCGAATCC		

[a], Primers also used for sequencing;

References for Table S1:

[1] Harper CG, Xu S, Rogers AB, Feng Y, Shen Z, Taylor NS et al. Isolation and characterization of novel *helicobacter* spp. From the gastric mucosa of harp seals *phoca groenlandica*. Dis Aquat Organ 2003; 57:1-9.

[2] Shen Z, Feng Y, Dewhirst FE, Fox JG. Coinfection of enteric *helicobacter* spp. And *campylobacter* spp. In cats. J Clin Microbiol 2001; 39:2166-72.

[3] Johnson JR, Johnston B, Kuskowski MA, Nougayrede JP, Oswald E. Molecular epidemiology and phylogenetic distribution of the *Escherichia coli* pks genomic island. J Clin Microbiol 2008; 46:3906-11.

[4] Dubois D, Delmas J, Cady A, Robin F, Sivignon A, Oswald E et al. Cyclomodulins in urosepsis strains of *Escherichia coli*. J Clin Microbiol 2010; 48:2122-29.

[5] Tóth I, Hérault F, Beutin L, Oswald E. Production of cytolethal distending toxins by pathogenic Escherichia coli strains isolated from human and animal sources: establishment of the existence of a new cdt variant (Type IV). J Clin Microbiol. 2003;41(9):4285-91

[6] Clermont O, Bonacorsi S, Bingen E. Rapid and simple determination of the *Escherichia coli* phylogenetic group. Appl Environ Microbiol 2000; 66:4555-58.

[7] Bidet P, Metais A, Mahjoub-Messai F, Durand L, Dehem M, Aujard Y et al. Detection and identification by pcr of a highly virulent phylogenetic subgroup among extraintestinal pathogenic *Escherichia coli* b2 strains. Appl Environ Microbiol 2007; 73:2373-77.

[8] Heininger A, Binder M, Schmidt S, Unertl K, Botzenhart K, Döring G. Pcr and blood culture for detection of *Escherichia coli* bacteremia in rats. J Clin Microbiol 1999; 37:2479-82.

Table S2. Virulence Genes

Isolates	Gene	Product	Virulence Function/Comments	References
S1, S2	*ast*A	Enteroaggregative E. coli (Eaggec) Heat-Stable Enterotoxin 1 (EAST1)	Activates guanylyl cyclase resulting in ion secretion<br>Possibly contributes to watery diarrheal disease	Croxen, M.A., 2010; Kaper, J.B. , 2004; Turner, S.M., 2006
S1, S2, S4, S5	*celb*	Endonuclease Colicin E2	Bacteriocin – DNA nuclease that translocates into susceptible E. coli strains and causes cell death via DNA damage<br>Hypothesized to provide competitive survival advantage colicin-expressing, pathogenic E. coli strains.<br>Mostly identified in B2 or D phylogroup	Alonso, G., 2000; Duche, D., 2007; Gordon, D.M., 2006; Lechner, M., et al., 2016; Micenkova, L., 2016
S1, S2, S3, S6, S9, S10	*cnf1*	Cytotoxic Nectrotizing Factor 1	Glutamine deaminase that targets Rho family GTPases (RhoA, Cdc42 and Rac) leading to constitutive “on” activity that causes aberrant actin cytoskeletal alterations, G2/M cell cycle arrest with multi-nucleation and cellular enlargement, and anti-apoptotic/pro-survival signaling pathways<br>Promotes persistence of epithelial colonization niches and invasion/dissemination of infection.<br>May contribute toward carcinogenesis	Buc, E., 2013; Croxen, M.A., 2010; Flores-Mireles, A.L., 2015; Kaper, J.B. , 2004
S1-S10	*gad*	Glutamate Decarboxylase	Promotes survival in acid environments by neutralizing cytoplasmic pH via decarboxylation of glutamate and export of γ-aminobutyric acid<br>May allow survival through stomach for intestinal colonization during transmission of infection	Bergholz, T.M., 2007; Grant, M.A., 2001; Shin, S., 2001
S3, S6, S9, S10	*iha*	Iron-Regulated Gene Homologue Adhesin	Adhesion factor – adherence to epithelial cells to promote colonization of host<br>Expression increased in iron depleted environments	Johnson, J.R., 2005; Rashid, R.A., 2006; Tarr, P.I., 2000
S1, S2, S4, S5	*iro*N	Enterobactin Siderophore Receptor Protein	Iron acquisition – transportation of iron-cHeLated siderophore into bacteria	Caza, M., 2013; Flores-Mireles, A.L., 2015; Kaper, J.B. , 2004
S1-S10	*iss*	Increased Serum Survival/Bor Protein Precursor	Outer membrane protein – promotes resistance against killing by host complement proteins<br>Possibly promote survival against innate immune system during systemic dissemination/infection	Johnson, T.J. , 2008; Lynne, A.M., 2007; Miajlovic, H., 2014
S7, S9	*lpf*A	Long Polar Fimbriae	Adhesion factor – adherence to intestinal epithelial cells to promote colonization of host	Jordan, D.M., 2004; Ross, B.N., et al., 2015; Torres, A.G., 2009
S1, S2, S4, S5	*mch*B, *mch*C, *mch*F, *mcm*A	Microcin H47 Synthesis and Transport	Bacteriocin - ribosomally-synthesized peptide antibiotic against susceptible bacterial species<br>Hypothesized to provide competitive survival advantage microcin -expressing, pathogenic E. coli strains.<br>Mostly identified in B2 or D phylogroup.<br>Association between microcin H47 and higher number of virulence genes in genome	Abraham, S., 2012; Budic, M., 2011; Micenkova, L., 2014.; Micenkova, L., 2016; Lavina, M., 1990; Rodriguez, E., 199o
S4, S5	*pic*	Protease Involved in Intestinal Colonization	Serine Protease autotransporters of Enterobacteriaceae (SPATE)<br>Mucinase activity may mediate degradation of mucus or mucin as a nutrient source and facilitate penetration/invasion through mucous barrier layer<br>Protease activity may cause serum resistance possibly by degradation of complement proteins	Croxen, M.A., 2010; Dautin, N., 2010; Kaper, J.B., 2004; Welch, R.A., 2016
S1, S2, S4, S5	pks Gene Island *(clb*A-*clb*R*)*	Colibactin	Genotoxin that causes G2/M cell cycle arrest, megalocytosis, and DNA damage to cell lines in vitro<br>Associated with colorectal cancer in human patients and potentiates carcinogenesis/tumor formation in mouse model	Arthur, J.C., 2012; Buc, E., 2013; García, A., 2016; Nougayrede, J.P., 2006
S1, S2, S4, S5	*vat*	Vacuolating Autotransporter Toxin	Serine Protease Autotransporters of Enterobacteriaceae (SPATE)<br>Vacuolating activity causes intracellular vacuoles to cell lines in vitro	Guignot, J., A., 2015; Welch, R.A., 2016
S3, S6, S9, S10	*sat*	Secreted Autotransporter Toxin	Serine Protease Autotransporters of Enterobacteriaceae (SPATE)<br>Cytotoxicity characterized by vacuolating activity, elongated morphology, plate detachment, and death to cell lines in vitro<br>May degrade tight junctions between intestinal epithelial cells to promote invasion	Dautin, N.,2010; Welch, R.A., 2016

References for Table S2:

1. Abraham, S., et al., Molecular characterization of Escherichia coli strains that cause symptomatic and asymptomatic urinary tract infections. J Clin Microbiol, 2012. 50(3): p. 1027-30.
2. Alonso, G., G. Vilchez, and V. Rodriguez Lemoine, How bacteria protect themselves against channel-forming colicins. Int Microbiol, 2000. 3(2): p. 81-8.
3. Arthur, J.C., et al., Intestinal inflammation targets cancer-inducing activity of the microbiota. Science, 2012. 338(6103): p. 120-3.
4. Bergholz, T.M., et al., Recent gene conversions between duplicated glutamate decarboxylase genes (gadA and gadB) in pathogenic Escherichia coli. Mol Biol Evol, 2007. 24(10): p. 2323-33.
5. Buc, E., et al., High Prevalence of Mucosa-Associated E. coli Producing Cyclomodulin and Genotoxin in Colon Cancer. PLoS One, 2013. 8(2).
6. Budic, M., et al., Escherichia coli bacteriocins: antimicrobial efficacy and prevalence among isolates from patients with bacteraemia. PLoS One, 2011. 6(12): p. e28769.
7. Caza, M. and J.W. Kronstad, Shared and distinct mechanisms of iron acquisition by bacterial and fungal pathogens of humans. Front Cell Infect Microbiol, 2013. 3: p. 80.
8. Croxen, M.A. and B.B. Finlay, Molecular mechanisms of Escherichia coli pathogenicity. Nat Rev Microbiol, 2010. 8(1): p. 26-38.
9. Dautin, N., Serine protease autotransporters of enterobacteriaceae (SPATEs): biogenesis and function. Toxins (Basel), 2010. 2(6): p. 1179-206.
10. Duche, D., Colicin E2 is still in contact with its receptor and import machinery when its nuclease domain enters the cytoplasm. J Bacteriol, 2007. 189(11): p. 4217-22.
11. Flores-Mireles, A.L., et al., Urinary tract infections: epidemiology, mechanisms of infection and treatment options. Nat Rev Microbiol, 2015. 13(5): p. 269-84.
12. García, A., et al., Cytotoxic Escherichia coli strains encoding colibactin colonize laboratory mice. Microbes and Infection.
13. Gordon, D.M. and C.L. O'Brien, Bacteriocin diversity and the frequency of multiple bacteriocin production in Escherichia coli. Microbiology, 2006. 152(Pt 11): p. 3239-44.
14. Grant, M.A., S.D. Weagant, and P. Feng, Glutamate Decarboxylase Genes as a Prescreening Marker for Detection of Pathogenic Escherichia coli Groups. Applied and Environmental Microbiology, 2001. 67(7): p. 3110-3114.
15. Guignot, J., A. Segura, and G. Tran Van Nhieu, The Serine Protease EspC from Enteropathogenic Escherichia coli Regulates Pore Formation and Cytotoxicity Mediated by the Type III Secretion System. PLoS Pathogens, 2015. 11(7): p. e1005013.
16. Johnson, J.R., et al., The IrgA homologue adhesin Iha is an Escherichia coli virulence factor in murine urinary tract infection. Infect Immun, 2005. 73(2): p. 965-71.
17. Johnson, T.J., Y.M. Wannemuehler, and L.K. Nolan, Evolution of the iss gene in Escherichia coli. Appl Environ Microbiol, 2008. 74(8): p. 2360-9.
18. Jordan, D.M., et al., Long polar fimbriae contribute to colonization by Escherichia coli O157:H7 in vivo. Infect Immun, 2004. 72(10): p. 6168-71.
19. Kaper, J.B., J.P. Nataro, and H.L. Mobley, Pathogenic Escherichia coli. Nat Rev Microbiol, 2004. 2(2): p. 123-40.
20. Lavina, M., C. Gaggero, and F. Moreno, Microcin H47, a chromosome-encoded microcin antibiotic of Escherichia coli. J Bacteriol, 1990. 172(11): p. 6585-8.
21. Lechner, M., et al., Hierarchical Post-transcriptional Regulation of Colicin E2 Expression in Escherichia coli. PLoS Comput Biol, 2016. 12(12): p. e1005243.
22. Lynne, A.M., et al., Detection of Iss and Bor on the surface of Escherichia coli. J Appl Microbiol, 2007. 102(3): p. 660-6.
23. Miajlovic, H. and S.G. Smith, Bacterial self-defence: how Escherichia coli evades serum killing. FEMS Microbiol Lett, 2014. 354(1): p. 1-9.
24. Micenkova, L., et al., Bacteriocin-encoding genes and ExPEC virulence determinants are associated in human fecal Escherichia coli strains. BMC Microbiol, 2014. 14: p. 109.
25. Micenkova, L., et al., Microcin determinants are associated with B2 phylogroup of human fecal Escherichia coli isolates. Microbiologyopen, 2016. 5(3): p. 490-8.
26. Nougayrede, J.P., et al., Escherichia coli induces DNA double-strand breaks in eukaryotic cells. Science, 2006. 313(5788): p. 848-51.
27. Rashid, R.A., P.I. Tarr, and S.L. Moseley, Expression of the Escherichia coli IrgA homolog adhesin is regulated by the ferric uptake regulation protein. Microb Pathog, 2006. 41(6): p. 207-17.
28. Rodriguez, E., C. Gaggero, and M. Lavina, The structural gene for microcin H47 encodes a peptide precursor with antibiotic activity. Antimicrob Agents Chemother, 1999. 43(9): p. 2176-82.
29. Ross, B.N., et al., The Role of Long Polar Fimbriae in Escherichia coli O104:H4 Adhesion and Colonization. PLoS One, 2015. 10(10): p. e0141845.
30. Shin, S., et al., An activator of glutamate decarboxylase genes regulates the expression of enteropathogenic Escherichia coli virulence genes through control of the plasmid-encoded regulator, Per. Mol Microbiol, 2001. 41(5): p. 1133-50.
31. Tarr, P.I., et al., Iha: a novel Escherichia coli O157:H7 adherence-conferring molecule encoded on a recently acquired chromosomal island of conserved structure. Infect Immun, 2000. 68(3): p. 1400-7.
32. Torres, A.G., et al., Genes related to long polar fimbriae of pathogenic Escherichia coli strains as reliable markers to identify virulent isolates. J Clin Microbiol, 2009. 47(8): p. 2442-51.
33. Turner, S.M., et al., Weapons of mass destruction: virulence factors of the global killer enterotoxigenic Escherichia coli. FEMS Microbiol Lett, 2006. 263(1): p. 10-20.
34. Welch, R.A., Uropathogenic Escherichia coli-Associated Exotoxins. Microbiol Spectr, 2016. 4(3).
