# Supplementary figures and images for: Cytotoxic Escherichia coli strains encoding colibactin and cytotoxic necrotizing factor (CNF) colonize laboratory macaques
Source: Gut Pathog. 2017 Dec 6;9:71. doi: 10.1186/s13099-017-0220-y (PMC5718112; doi:10.1186/s13099-017-0220-y)

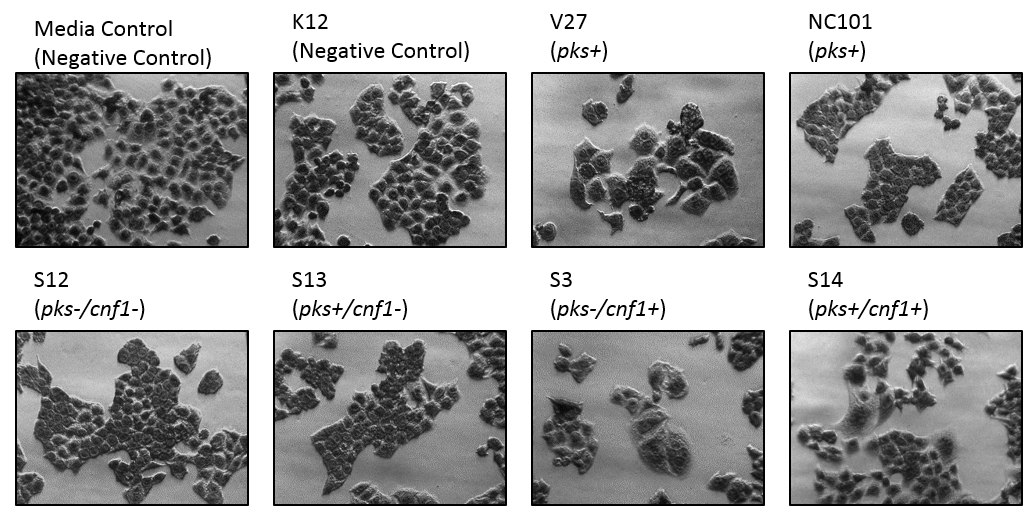

Supplement: Supplementary file 2 — Additional file 2: Figure S1. Only treatment with supernatant from the cnf1-encoding novel rhesus macaque isolates (S3, S14) caused cell body enlargement and multi-nucleation. No cytotoxicity was observed after supernatant treatment with the other novel isolates. Images were taken at 20× magnification. [file 13099_2017_220_MOESM2_ESM.tif]
